# Low serum parathyroid hormone is a risk factor for peritonitis episodes in incident peritoneal dialysis patients: a retrospective study

**DOI:** 10.1186/s12882-021-02241-0

**Published:** 2021-01-29

**Authors:** Yuqi Yang, Jingjing Da, Yi Jiang, Jing Yuan, Yan Zha

**Affiliations:** 1 Renal Division, Department of Medicine, Guizhou Provincial People’s Hospital, Guiyang, China; 2 NHC Key Laboratory of Pulmonary Immunologic Disease, Guizhou Provincial People’s Hospital, Guiyang, China; 3 Information section, Provincial People’s Hospital, Guiyang, China

**Keywords:** Parathyroid hormone, Peritonitis, Peritoneal dialysis

## Abstract

**Background:**

Serum parathyroid hormone (PTH) levels have been reported to be associated with infectious mortality in peritoneal dialysis (PD) patients. Peritonitis is the most common and fatal infectious complication, resulting in technique failure, hospital admission and mortality. Whether PTH is associated with peritonitis episodes remains unclear.

**Methods:**

We examined the association of PTH levels and peritonitis incidence in a 7-year cohort of 270 incident PD patients who were maintained on dialysis between January 2012 and December 2018 using Cox proportional hazard regression analyses. Patients were categorized into three groups by serum PTH levels as follows: low-PTH group, PTH < 150 pg/mL; middle-PTH group, PTH 150-300 pg/mL; high-PTH group, PTH > 300 pg/mL.

**Results:**

During a median follow-up of 29.5 (interquartile range 16–49) months, the incidence rate of peritonitis was 0.10 episodes per patient-year. Gram-positive organisms were the most common causative microorganisms (36.2%), and higher percentage of Gram-negative organisms was noted in patients with low PTH levels. Low PTH levels were associated with older age, higher eGFR, higher hemoglobin, calcium levels and lower phosphate, alkaline phosphatase levels. After multivariate adjustment, lower PTH levels were identified as an independent risk factor for peritonitis episodes [hazard ratio 1.643, 95% confidence interval 1.014–2.663, *P* = 0.044].

**Conclusions:**

Low PTH levels are independently associated with peritonitis in incident PD patients.

## Background

Abnormalities in serum parathyroid hormone (PTH) are exceedingly common in end-stage renal disease (ESRD) patients on maintenance dialysis and associated with cardiovascular disease, disturbances in bone mineral disorders, even increased morbidity and mortality in most epidemiologic studies [1, 2]. The role of PTH has been investigated as a traditional biomarker of chronic kidney disease-mineral bone disorder (CKD-MBD) in dialysis patients for decades [2, 3]. However, PTH, influenced by age, inflammation and nutrition [4], is also an important factor influencing immunologic dysfunction for infectious diseases, which are important causes of mortality in dialysis patients. More attention has been attracted to an increased understanding of the role of PTH in inflammation status that extends beyond MBD. A recent study demonstrated that PTH could be a pro-inflammatory parameter independent from the degree of renal dysfunction [5]. Low PTH levels can induce inflammation, malnutrition and protein-energy wasting [6], besides, inflammation can induce suppression of PTH secretion [7]. Dukkipati et al. [8] have proven an association between low PTH levels (< 150 pg/mL) and malnutrition-inflammation-complex (MICS) in dialysis patients. In a large prospective cohort of 1771 incident dialysis patients, Hong et al. [9] identified that low PTH levels (< 150 pg/mL) were an independent risk factor for infection-related mortality in dialysis patients both with hemodialysis and with PD and was even more meaningful in infection-related mortality than all-cause mortality.

PD-related peritonitis remains the most major and life-threatening infection-related complication and is closely related to loss of catheter function, impairment of the peritoneal membrane, eventually discontinuation of PD therapy, conversion to hemodialysis [10, 11]. The 2016 International Society for Peritoneal Dialysis (ISPD) guidelines recommend a benchmark of 0.5 episodes per year or one episode every 2 years [12]. Peritonitis results in an overall mortality rate of up to 15% of PD patients [13]. Risk factors associated with peritonitis, including older age, diabetes, hypoalbuminemia [14, 15], also reflect a status of malnutrition in PD patients. Therefore, we suspect that low serum PTH levels may play a role in incidence of PD-related peritonitis. To our knowledge, there are no data available about the association of PTH with peritonitis in PD patients.

This study was carried out to estimate the association of serum PTH levels with PD-related peritonitis. We hypothesized that lower PTH might be associated with higher incidence rate of peritonitis in PD patients.

## Methods

### Study population

This was a single-center and retrospectively designed study. Patients older than 18 years old who started maintenance PD in the PD clinic of Guizhou Provincial People’s Hospital between 1 January 2012 and 31 December 2018 were recruited in the study. Patients with PD treatment< 3 months, unavailable data of baseline PTH levels, and lack of proper follow up were excluded. Accordingly, the final study consisted of 270 incident PD patients. The study fulfilled the ethic requirements of Guizhou Provincial People’s Hospital’s institutional committee on human experimentation for observational, retrospective studies and complied with the principles of the Declaration of Helsinki for medical research.

### Patient characteristics

All data were obtained from the electronic medical records of dialysis facilities. A review of basic demographic and clinical variables of the patients, including age, sex, body mass index (BMI), primary cause of ESRD, presence of diabetes mellitus, systolic and diastolic blood pressure levels, was undertaken. The primary renal diseases were composed of the following classes: glomerulonephritis, diabetes mellitus, vascular renal disease, polycystic kidney disease, uncertain aetiology and others. BMI was calculated as weight/height^2^(kg/m^2^). Residual renal function expressed as estimated glomerular filtration rate (eGFR) was analyzed using the simplified Modification of Diet in Renal Disease (MDRD) study equation [16].

The laboratory parameters within three months after initiation of PD, as baseline of this study, were collected. These are white blood cell, neutrophil, lymphocyte, platelet counts and hemoglobin levels; serum total protein, albumin, urea nitrogen, creatinine and urine acid; calcium, phosphorus, alkaline phosphatase and PTH; total cholesterol and triglyceride; C-reactive protein levels. The type of causative organisms for patients at the first peritonitis episode were collected. All PD patients had the surgery in our center and the prophylactic antibiotic had been given before and after surgery routinely. The antibiotic cream was used for exit care during PD therapy. All PD therapies were done using a twin-bag with conventional PD solution for four or five times a day. All PD patients used glucose-containing PD fluid with 1.5% or 2.5% concentration according the dialysis effect. All patients who had secondary hyperparathyroidism were treated with oral active vitamin D according to the clinical practice guidelines from the Kidney Disease Improving Global Outcomes (KDIGO) (serum PTH level > 300 pg/mL, phosphorus < 1.78 mmol/L, calcium< 2.37 mmol/L) [17]. Vitamin D therapy after initiation of PD was recorded.

### Exposure and endpoints

The primary exposure was PTH during PD treatment. All patients had a baseline intact PTH measurement with a standard enzyme-linked immunosorbent assay-PTH immunoradiometric assay. In all analyses, PTH was categorized into three groups: < 150 pg/mL, 150-300 pg/mL, > 300 pg/mL. The categories and the reference of PTH (150-300 pg/mL) were chosen, according to the recommendation of 2016 ISPD. Patients were followed up from the initiation of PD until withdrawal from PD or the end period on 31 December 2019.

### Related definitions

Peritonitis was diagnosed if at least two of the following were present: (1) clinical features consistent with peritonitis (e.g. abdominal pain and/or cloudy effluent), (2) dialysis effluent white cell count > 100 cells/uL, with > 50% polymorphonuclear leukocytes, and (3) positive dialysis effluent culture [12].

### Statistical analyses

Continuous variables were expressed by mean values with standard deviation (SD) if normally distributed or median (first and third quartiles) if not normally distributed and categorical variables by frequencies and percentages. The normality of distribution for continuous variables was confirmed with the Kolmogorov-Smirnov test. For comparison among different categories of PTH, Chi-squared, one-way ANOVA, or Kruskal-Wallis tests were used as appropriate. Stepwise logistic regression analyses were applied to explore the association of low PTH with other clinical characteristics. Kaplan-Meier survival was used to compare peritonitis-free times, and the log-rank statistic was used to test differences among three groups. We aimed to determine the prognostic value of PTH level for the incidence of peritonitis. Univariate Cox proportional hazards model was used to assess clinical parameters which were associated with peritonitis. We then used a multivariate Cox regression hazards model including all significant factors from univariate analysis. Odds ratios (ORs), risk ratios (RRs) and their 95% CI were also calculated. Low PTH levels were defined as those that were lower than the ISPD-recommended PTH levels, which were between two- and night- times the upper limit of the normal PTH levels. Therefore, we chose 150 pg/mL as the cutoff value for low PTH levels. A two-tailed *P* value< 0.05 was considered to indicate a statistically significant difference. SPSS version 23.0 (IBM Corp., Armonk, NY, USA) was employed for all statistical analyses.

## Results

### Patient characteristics

Table 1 shows the baseline clinical characteristics of PD patients. In total, 315 ESRD patients who started maintenance PD therapy for the first time between 1 January 2012 and 31 December 2018 were followed-up at our PD center, of whom 7 patients were younger than 18 years, 12 patients were on PD less than 3 months, and 26 patients had not available data of baseline PTH levels. The remaining 270 patients were enrolled in this study. The mean (±SD) age was 39.9 ± 13.3 years, 55.9% of patients were male, and 8.9% of patients were diabetic. The primary cause of ESRD was primary glomerulonephritis (71.5%) followed by diabetes nephropathy (8.9%) and hypertension (11.1%). It’s worth mentioning that eight patients whose primary cause of ESRD was vasculitis have not received immunosuppressive treatment, and have initiated PD therapy because of a low disease activity.

**Table 1:** Baseline characteristics of 270 PD patients by PTH categories

Characteristic	Total (*n* = 270)	Baseline PTH levels (pg/mL)			*P*-value
		< 150 (n = 78)	150–300 (n = 69)	> 300 (*n* = 123)	
Age(years)	39.9 ± 13.3	44.0 ± 14.0	37.8 ± 11.6	38.6 ± 13.2	0.005
Male (n,%)	151(55.9%)	47(60.3%)	35(50.7%)	61(50.4%)	0.307
BMI (kg/m^2^)	21.6 ± 3.9	21.8 ± 3.9	21.4 ± 4.0	21.6 ± 3.8	0.814
Primary cause of ESRD					
Glomerulonephritis	193(71.5%)	48(61.5%)	53 (76.8%)	92(74.8%)	0.067
Diabetic kidney disease	24(8.9%)	12(15.4%)	7(10.1%)	5(4.1%)	0.022
Hypertensive kidney disease	30(11.1%)	8(10.3%)	6(8.7%)	16(13.0%)	0.633
Vasculitis kidney disease	8(3.0%)	5(6.4%)	1(1.4%)	2 (1.6%)	0.151
Others	21(7.8%)	6(7.7%)	5(7.2%)	10(8.1%)	0.976
SBP (mmHg)	141.4 ± 19.7	137.5 ± 18.6	143.3 ± 20.8	142.7 ± 19.5	0.187
DBP (mmHg)	91.6 ± 15.7	88.8 ± 14.2	94.3 ± 17.0	91.8 ± 15.5	0.158
White blood cell (× 10^9^/L)	6.6 ± 2.1	6.9 ± 2.2	6.5 ± 2.2	6.5 ± 1.9	0.220
Neutrophil (× 10^9^/L)	4.5 ± 1.7	4.8 ± 1.8	4.6 ± 2.0	4.4 ± 1.5	0.321
Lymphocyte (×10^9^/L)	1.5 ± 0.5	1.6 ± 0.6	1.4 ± 0.4	1.4 ± 0.5	0.103
Hemoglobin (g/L)	100.8 ± 22.0	107.3 ± 21.7	98.3 ± 21.7	98.1 ± 21.6	0.008
Platelet (×10^9^/L)	195.7 ± 74.9	202.1 ± 73.0	205.3 ± 83.9	186.3 ± 70.1	0.160
Urea nitrogen (mmol/L)	14.9 ± 7.6	13.7 ± 7.3	15.4 ± 8.1	15.4 ± 7.4	0.272
Creatinine (umol/L)	764.1(590.4–1017.4)	701.9(539.3–909.7)	786.8(628.3–1094.6)	791.7(613.2–1084.6)	0.023
eGFR (/kg/m^2^)	8.1 (6.0–10.3)	9.2(6.7–11.3)	7.7(6.3–9.4)	7.8(5.7–9.9)	0.069
Uric acid (umol/L)	421.1 ± 108.8	420.3 ± 95.9	423.3 ± 116.1	420.3 ± 112.9	0.981
Calcium (mmol/L)	2.2 ± 0.2	2.3 ± 0.2	2.2 ± 0.2	2.1 ± 0.2	0.000
Phosphate (mmol/L)	1.4 ± 0.5	1.3 ± 0.5	1.5 ± 0.4	1.5 ± 0.5	0.001
PTH (pg/mL)	260.05 (121.7–447.0)	74.5(28.3–109.9)	226.7(185.8–258.9)	475.3(364.0–694.7)	0.000
Alkaline phosphatase (U/L)	74.0(60.0–98.5)	64.0(56.0–83.3)	70.0(61.5–89.5)	82.5 (68.0–122.5)	0.000
Cholesterol (mmol/L)	4.7 ± 1.1	4.8 ± 1.2	4.8 ± 1.2	4.7 ± 1.0	0.788
Triglyceride (mmol/L)	1.7 ± 0.9	1.9 ± 1.1	1.6 ± 0.8	1.6 ± 0.8	0.063
Total protein(g/L)	64.1 ± 8.5	64.4 ± 9.5	63.8 ± 6.9	64.1 ± 8.7	0.925
Albumin(g/L)	35.3 ± 6.0	35.9 ± 6.7	35.4 ± 5.2	34.9 ± 6.0	0.519
C-reactive protein(mmol/L)	1.17(0.61,4.91)	2.02(0.61,11.2)	1.1(0.5,6.2)	1.1(0.7,4.9)	0.768
Active vitamin D use (n, %)	161(59.6%)	25(32.1%)	34(49.3%)	102(82.9%)	0.000

Data are presented as mean ± SD, median (interquartile ranges) or percentages

*Abbreviations: BMI* body mass index, *ESRD* end stage renal disease, *SBP* systolic blood pressure, *DBP* diastolic blood pressure, *eGFR* estimated glomerular filtration rate, *PTH* parathyroid hormone

All enrolled patients were divided into three groups: low-PTH group (*n* = 78), middle-PTH group (*n* = 69) and high-PTH group (*n* = 123). Differences in baseline characteristics were observed between patients with different PTH levels. PD patients with low serum PTH levels were significantly older, with a higher proportion of diabetes mellitus as the primary cause of ESRD. Moreover, lower PTH patients tended to have higher levels of hemoglobin, calcium and lower levels of creatinine, phosphate, alkaline phosphatase at baseline.

### Association of PTH levels with clinical parameters

Table 2 shows the association of PTH with clinical parameters. In univariate logistic regression analysis, the variables that were associated with an increased risk of PTH < 150 pg/mL included older age (OR = 0.968, *P* = 0.002), diabetes mellitus (OR = 0.367, *P* = 0.020), high hemoglobin (OR = 0.981, *P* = 0.003), eGFR (OR = 0.925, *P* = 0.014), calcium (OR = 0.040, *P* = 0.000), triglyceride (OR = 0.718, *P* = 0.029), levels, low phosphate (OR = 3.586, P = 0.000), alkaline phosphatase (OR = 1.017 P = 0.003). In a multivariate logistic regression model, older age (OR = 0.924, *P* = 0.002), higher hemoglobin (OR = 0.980, *P* = 0.026), eGFR (OR = 0.913, *P* = 0.009), calcium levels (OR = 0.018, *P* = 0.018), lower phosphate (OR = 1.084, *P* = 0.034), alkaline phosphatase levels (OR = 1.005, *P* = 0.020) remained statistically significant.

**Table 2:** Logistic regression of associated with low serum PTH levels (< 150 pg/mL)

Clinical factors	Univariate analysis				Multivariate analysis			
	OR	95%CI		P value	OR	95%CI		P value
		lower	upper			lower	upper	
Age, years	0.968	0.949	0.988	0.002	0.953	0.924	0.982	0.002
Diabetes mellitus	0.367	0.157	0.857	0.020	0.538	0.134	2.154	0.381
White blood cell	0.895	0.790	1.015	0.084	1.026	0.831	1.267	0.811
Hemoglobin	0.981	0.969	0.993	0.003	0.980	0.963	0.997	0.026
eGFR	0.925	0.870	0.984	0.014	0.913	0.853	0.977	0.009
Calcium	0.040	0.011	0.151	0.000	0.018	0.003	0.127	0.018
Phosphate	3.586	1.769	7.267	0.000	2.946	1.084	8.002	0.034
Triglyceride	0.718	0.534	0.966	0.029	0.768	0.495	1.190	0.238
Alkaline phosphatase	1.017	1.006	1.028	0.003	1.020	1.005	1.036	0.010

*Abbreviations: eGFR* estimated glomerular filtration rate

### PD-associated peritonitis incidence

**Fig. 1:**
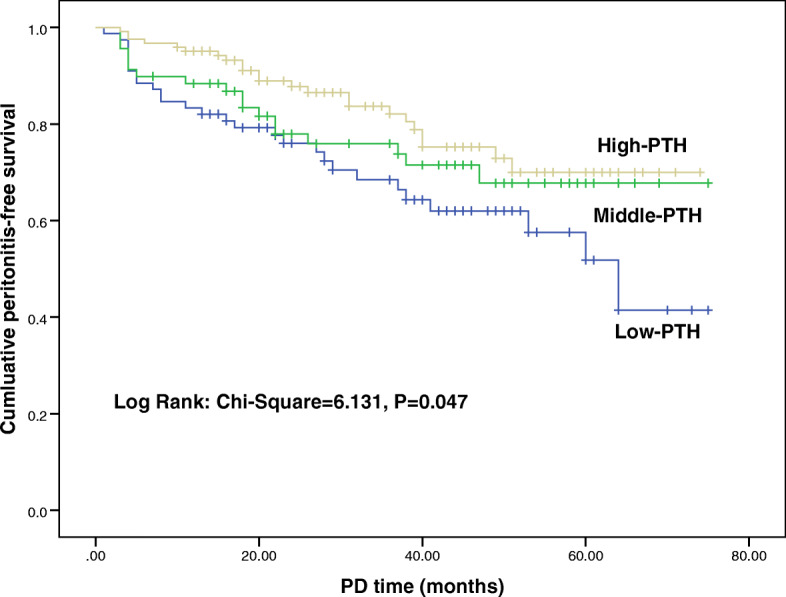
Kaplan-Meier curves for time to first episode of peritonitis

Table 3 shows the incidence of peritonitis during the follow-up period. After a 7-years of follow-up (median follow-up duration: 29.5 months; interquartile range: 16 to 49 months), 69 (25.6%) patients had at least one episode of peritonitis and 201 (74.4%) patients experienced no episode of peritonitis. The overall incidence rate of peritonitis was 0.10 episodes/patient-year, the patients with low PTH levels had significantly more peritonitis [0.18 vs 0.07 vs 0.06 episodes/person-year, respectively (*P* = 0.000)] than those with middle, high PTH levels. Meanwhile, 25 (9.3%) patients had relapsed peritonitis, and patients in low-PTH group had the highest incidence rate of relapsed peritonitis (16.7% vs 10.1% vs 4.1%, P = 0.000). Figure 1 shows the peritonitis-free period for patients with different PTH levels during 7-year follow-up in the Kaplan-Meier analyses. Compared with other two groups, the median time to the first episode of peritonitis was significantly shorter in low-PTH group (Log rank test, *P* = 0.047).

**Table 3:** Incidence of PD-associated peritonitis by PTH categories

Characteristic	Total (n = 270)	Baseline PTH levels (pg/mL)			P-value
		< 150 (n = 78)	150–300 (n = 69)	> 300 (n = 123)	
PD duration (months)	29.5(16.0,49.0)	28.0(15.5,49.0)	37.0(16.5,49.5)	30.0(16.0,49.0)	0.810
Total patient-year(year)	739.3	206.2	194.8	338.3	0.786
Peritonitis (total number of episodes)	69(25.6%)	32(41.0%)	13(18.8%)	24(19.5%)	0.000
Incidence of peritonitis(/patient-year)	0.10	0.18	0.07	0.06	0.000
Relapsed peritonitis (n,%)	25(9.3%)	13(16.7%)	7(10.1%)	5(4.1%)	0.000

### PTH level and PD-associated peritonitis

In a univariate Cox proportional hazards model the following parameters were associated with an increased risk for peritonitis: higher age (> 65 years), diabetes mellitus, low serum PTH levels. We then used a multivariate Cox regression hazards model including all significant factors from univariate analysis. Low PTH levels (< 150 pg/mL) were significantly associated with a 1.643-fold increased risk of peritonitis incidence [HR 1.643 (95%CI 1.014–2.663), *P* = 0.044] (Table 4).

**Table 4:** Cox regression analysis of risk factors for time to first peritonitis

Clinical factors	Univariate analysis				Multivariate analysis			
	HR	95%CI		P value	HR	95%CI		P value
		lower	upper			lower	upper	
Age(years)								
≥ 65	1.023	1.005	1.041	0.013	0.600	0.247	1.458	0.259
< 65	1				1			
Diabetes mellitus(yes:no)	0.451	0.230	0.883	0.020	1.604	0.779	3.305	0.200
PTH(< 150 pg/mL)								
150	1.742	1.007	2.817	0.024	1.643	1.014	2.663	0.044
≥ 150	1				1			

*Abbreviations: PTH* parathyroid hormone

Multivariate analysis was adjusted for age, diabetes mellitus, PTH

### Causative microorganisms of PD-associated peritonitis

Table 5 demonstrates the causative microorganisms of first peritonitis episode in three groups. Gram-positive organisms were the most common in all pathogens (36.2%, being the *Staphylococcus aureus* the most common gram-positive species), followed by Gram-negative organisms (20.3%, being the *Escherichia coli* the most common gram-negative species). Higher percentage of Gram-negative organisms was noted in low-PTH group (28.1% vs 23.1% vs 8.3%, *P* = 0.000), in contrast, lower percentage of Gram-positive organisms was noted in low-PTH group (31.3% vs 38.5% vs 41.7%, *P* = 0.013).

**Table 5:** Causative organism characteristics of 69 patients with peritonitis by PTH categories

Causative microorganisms (n, %)	Total (n = 69)	Baseline PTH levels (pg/mL)			P-value
		< 150 (*n* = 32)	150–300 (*n* = 13)	> 300 (*n* = 24)	
Gram-positive organism	25(36.2%)	10(31.3%)	5(38.5%)	10(41.7%)	0.013
Staphylococcus sp.	17(24.6%)	4(12.5%)	5(38.5%)	8(33.3%)	0.000
*Staphylococcus aureus*	12(17.4%)	3(9.4%)	4(30.8%)	5(20.8%)	0.000
*Staphylococcus epidermidis*	3(4.3%)	1(3.1%)	0	2(8.3%)	–
MRSA	2(2.9%)	0	1(7.7%)	1(4.2%)	–
Streptococcus sp.	4(5.8%)	3(9.4%)	0	1(4.2%)	–
Enterococcus sp.	1(1.4%)	1(3.1%)	0	0	–
Others	3(4.3%)	2(6.3%)	0	1(4.2%)	–
Gram-negative organism	14(20.3%)	9(28.1%)	3(23.1%)	2(8.3%)	0.000
*Pseudomonas aeruginosa*	1(1.4%)	1(3.1%)	0	0	–
Esherichia coli	10(14.5%)	6(18.8%)	3(23.1%)	1(4.2%)	0.000
Others	3(4.3%)	2(6.3%)	0	1(4.2%)	–
Fungi	2(2.9%)	1(3.1%)	0	1(4.2%)	–
Culture-negative	28(40.6%)	12(37.5%)	5(38.5%)	11(45.8%)	0.036

*Abbreviations: PTH* parathyroid hormone, *MRSA* methicillin-resistant Staphylococcus aureus

## Discussion

This present study demonstrated that lower baseline PTH levels were associated with a greater risk of peritonitis. The overall incidence rate of peritonitis was nearly 1.6-fold greater in PD patients with low PTH levels than in the comparison cohort. In addition, low PTH levels were significantly associated with older age, high serum calcium levels and low alkaline phosphatase levels. To the best of our knowledge, we report for the first time that low PTH levels can be a potential predictor of peritonitis in PD patients.

In this cohort of PD patients over a 7-year follow-up, the prevalence of low PTH (< 150 pg/mL) levels was 28.9%, which was similar to that reported from the Dialysis Outcomes and Practice Patterns Study (DOPPS) that was undertaken in the United States in which 29% patients had PTH levels of < 100 pg/mL [18], and the United Kingdom Renal Registry (UKRR) study in which 32% of the patients had PTH levels of < 150 pg/mL [19]. In dialysis patients, low PTH levels play a vital role in influencing immunologic dysfunction for infectious diseases. PD patients have high infection rate due to the continuous peritoneal exposure to peritoneal dialysate and PD catheter [20]. In a recent study, Hong et al. [9] suggested that low PTH levels were risk factors of infection-related mortality in a cohort of 1771 incident dialysis patients, including 511 PD patients. Our study expanded the previous findings of the associations of PTH levels with infection-related diseases in PD patients. This study focused on the incidence of PD-related peritonitis, which was the most common and fatal infection-related implication of PD. A recent cohort study covering 1321 PD patients demonstrated that peritonitis was independently associated with near 2-fold increased risk of all-cause mortality and near 4-fold increased risk of infection-related mortality [21]. The results of the present study suggested that PD patients with lowest PTH levels had the highest incidence rates of peritonitis, and patients with highest PTH levels had the lowest incidence rates of peritonitis, which were in accordance with the associations of infectious mortality in the previous study.

The pathophysiologic mechanisms, how low PTH levels increase the susceptibility of peritonitis incidence in PD patients, are not still well explained. However, some explanations can be proposed.

On the one hand, the impairment of the immune system in PD patients leads to an increased incidence of infections, and PTH plays an important role in the long-term immunodeficient and inflammation state, which is the main contributor to the peritonitis episodes [22]. Previous studies in vitro and in vivo verified that PTH can stimulate the proliferation of T lymphocytes, due to its ability to augment the movement of calcium into its target cells and enhance the production of interleukin-2 [23]. Conversely, inflammation can induce the inhibition of the hormone through proinflammatory cytokines, including interleukin-1 and interleukin-6 [7, 24]. Inactivation of PTH will abolish its effects on T cells. Ozdemir et al. [25] observed that low PTH levels were associated with an inadequate suppression of inflammation through impairing humoral and cellular immune response [26]. Low PTH levels have been demonstrated to be a predictor of chronic inflammation in CKD patients, especially with maintenance dialysis. A study of 748 dialysis patients implicated that PTH < 150 pg/mL was associated with elevated inflammatory markers, including tumor necrosis factor α and C-reactive protein, which were traditional evaluated parameters for peritonitis [8]. In this study, C-reactive protein levels in low-PTH group were highest among three groups, though without statistical significance. Meanwhile, low PTH levels were associated with advanced age, likely to be diabetic in this study, which can reflect clinical susceptibility to infection.

Vitamin D deficiency, a common metabolic disturbance among PD patients [27], has been found to be independently predictive of increased risk of peritonitis in PD patients [28], due to its contribution to microinflammation and a defective immune system. Some previous studies also demonstrated that oral active vitamin D therapy was associated with a reduced risk of peritonitis through the improvement of the bactericidal response. Kerschbaum et al. [29] found that a 57% risk of peritonitis could be decreased by treatment with oral active vitamin D at a multivariate analysis from 726 PD patients. This study just collected the vitamin D therapy at the initiation of PD, but not the adjustment of vitamin D therapy during the PD duration. Therefore, there is no similar finding above in this study. The association between the variation of PTH levels, vitamin D therapy and peritonitis episodes need more studies searched.

On the other hand, low PTH levels can reflect a poor nutritional state, which will decline immunity and resistance to pathogens. Low immunity provides survival environment for conditional bacteria, inducing the episodes of peritonitis. As previous studies, we observed that patients with low PTH levels (< 150 pg/mL) had worse nutritional status including advanced age, diabetes mellitus, low serum creatinine, and phosphorus levels [30]. Inflammatory cytokines will increase due to immunodeficient status, and suppress PTH secretion further in a vicious circle. Fukagawa et al. [6] demonstrated that low PTH levels reflected a state of malnutrition in dialysis patients. Dukkipati et al. [8] advanced that low PTH levels might be a facet of the MICS, which per se was associated with chronic inflammation. In the foregoing study, patients with PTH < 150 pg/mL had higher malnutrition-inflammation score, a constellation of markers of malnutrition and inflammation, and the inflammation-induced suppression of PTH can be overcome by treatment of MICS. PD-related peritonitis may be the consequence of MICS. Meanwhile, low PTH levels were a predictor of protein-energy wasting, which also shared similar risk factors of inflammation in PD patients [4].

In addition, the interaction of microbiome with renal function is known as the gut-kidney axis. Uremic toxins originated by gut microbiota also affect the composition of gut microbiota and involve in inflammation state in dialysis patients [31]. Su et al. [32] demonstrated that intestinal symptoms were associated with increased risk for enteric peritonitis episodes. Mirzaeian et al. [33] assessed the effect of synbiotics in dialysis patients, which could improve intestinal environment and decrease intestinal concentration of nitrogenous metabolites. They identified that synbiotic therapy reduced inflammation and renal insufficiency, along with a parallel increase of PTH levels. In this study, we found that the predominant causative organism of peritonitis patients with low PTH levels was Gram-negative organism, especially *Esherichia coli*. It means that PD patients with low PTH levels may be especially vulnerable to peritonitis of enteric origin. We speculated the association of low PTH and impaired gut environment. Low PTH levels usually imply diet restriction in dialysis patients, aggravating the intestinal flora imbalance. As a result, increased bacterial translocation from the intestinal lumen into the peritoneal cavity provokes peritonitis of enteric origin [34]. To clarify this mechanism, further studies are needed.

The strengths of our study include its examination of a relatively large cohort of incident PD patients. And, we demonstrated the relationship between PTH and incidence of peritonitis in PD patients.

However, several limitations of our study should be discussed. First, the present findings were observational in nature, which precluded conclusions concerning causality. Second, the study population was restricted to patients in a single PD center of southwest China, which might limit the generalizability of the results to other ethnic groups. Third, we did not assess other biochemical markers related to CKD-MBD, such as fibroblast growth factor-23, vitamin D metabolites. Forth, we did lack the information of the prognosis of peritonitis episodes. Fifth, we lacked the information of the non-PD associated infections and responding antibiotic therapies during our observation period.

## Conclusions

The present study demonstrates that low PTH levels are independently associated with a higher risk of peritonitis incidence in incident PD patients. Our results support the importance of maintaining proper PTH levels. Physicians should pay more attention to the predictive value of PTH on infectious events in PD patients. Future large-scale studies should afford insights for understanding the potential mechanisms how PTH levels predict peritonitis incidence.

## Data Availability

The datasets used and analyzed in this study are available from the first author and corresponding author on reasonable request.

## Abbreviations

PTH: Parathyroid hormone
PD: Peritoneal dialysis
ESRD: End-stage renal disease
CKD-MBD: Chronic kidney disease-mineral bone disorder
MICS: Malnutrition- inflammation-complex syndrome
ISPD: International Society for Peritoneal Dialysis
BMI: Body mass index
eGFR: Estimated glomerular filtration rate
MDRD: Modification of Diet in Renal Disease
SBP/DBP: Systolic/diastolic blood pressure
